# Viscoelasticity of PPA/SBS/SBR Composite Modified Asphalt and Asphalt Mixtures Under Pressure Aging Conditions

**DOI:** 10.3390/polym17050698

**Published:** 2025-03-06

**Authors:** Zongjie Yu, Xinpeng Ling, Ze Fan, Yueming Zhou, Zhu Ma

**Affiliations:** 1 College of Transportation Engineering, Changsha University of Science and Technology, Changsha 410114, China; 2 Xinjiang Institute of Transportation Sciences Co., Ltd., Urumqi 830000, China; xinpenglingxjk@163.com (X.L.); zefanxjk@163.com (Z.F.); yuemingzhou2024@163.com (Y.Z.); 3 Key Laboratory of Highway Engineering Technology and Transportation Industry in Arid Desert Regions, Urumqi 830000, China; 4 Xinjiang Highway and Bridge Testing and Inspection Center Co., Ltd., Urumqi 830000, China; zhumajczx@163.com

**Keywords:** road engineering, PPA/SBS/SBR composite modified asphalt mixture, FTIR, GPC, viscoelasticity, dynamic modulus

## Abstract

The viscoelastic behavior of asphalt mixtures is a crucial consideration in the analysis of pavement mechanical responses and structural design. This study aims to elucidate the molecular structure and component evolution trends of polyphosphoric acid (PPA)/styrene butadiene styrene block copolymer (SBS)/styrene butadiene rubber copolymer (SBR) composite modified asphalt (CMA) under rolling thin film oven test (RTFOT) and pressure aging (PAV) conditions, as well as to analyze the viscoelastic evolution of CMA mixtures. First, accelerated aging was conducted in the laboratory through RTFOT, along with PAV tests for 20 h and 40 h. Next, the microscopic characteristics of the binder at different aging stages were explored using Fourier-transform infrared spectroscopy (FTIR) and gel permeation chromatography (GPC) tests. Additionally, fundamental rheological properties and temperature sweep tests were performed to reveal the viscoelastic evolution characteristics of CMA. Ultimately, the viscoelastic properties of CMA mixtures under dynamic loading at different aging stages were clarified. The results indicate that the incorporation of SBS and SBR increased the levels of carbonyl and sulfoxide factors while decreasing the level of long-chain factors, which slowed down the rate of change of large molecule content and reduced the rate of change of LMS by more than 6%, with the rate of change of overall molecular weight distribution narrowing to below 50%. The simultaneous incorporation of SBS and SBR into CMA mixtures enhanced the dynamic modulus in the 25 Hz and −10 °C range by 24.3% (AC-13), 15.4% (AC-16), and reduced the φ by 55.8% (AC-13), 40% (AC-16). This research provides a reference for the application of CMA mixtures in the repair of pavement pothole damage.

## 1. Introduction

Asphalt aging is one of the factors leading to the deterioration of pavement performance [1]. Currently, researchers have begun to explore the effects of asphalt aging on its performance through numerous laboratory experiments [2,3]. Studies have demonstrated that the complex modulus of SBS modified asphalt increases with aging time while the phase angle decreases [4,5]. This trend results in reduced adhesion and the onset of stress relaxation in SBS modified asphalt. The study found that the addition of polymers can achieve three effects: first, it reduces the problem of surface raveling; second, it slows down the aging rate of the matrix asphalt; and third, it enhances the aging performance of the polymer-modified asphalt [6,7,8,9].

During the aging process of asphalt, short-chain compounds participate in addition and polymerization reactions, resulting in the formation of longer-chain compounds. The transformation of aromatic fractions and resins into asphalt contributes to the leading to asphalt hardening and embrittlement of the material [10,11]. Modified asphalt obtained by incorporating SBS and SBR into the base asphalt is used due to its excellent performance [12]. The presence of carbonyl compounds and sulfur oxides in aged asphalt contributes to a significant reduction in the effectiveness of SBS and SBR modifiers. Aged modified asphalt exhibits increased hardness and a notable decrease in low-temperature ductility, which reflects the combined effects of oxidation of asphalt molecules and degradation of compounds [11,13,14,15].

The inclusion of suitable components can enhance both high-temperature performance and cohesiveness [16]. Polystyrene can improve the low-temperature performance and adhesion of the CMA [17], while polyurethane enhances water stability [18], PPA improved the SBS modified asphalt’s resistance to permanent deformation and enhanced their elastic recovery properties [19,20]. After adding PPA, the penetration and ductility of composite modified asphalt were reduced, while the softening point, rutting factor and viscosity increased [21]. PPA prompted the clustering of SBS particles and confined the swelling of SBS [22]. The SBS/SBR modified asphalts with the addition of PPA show significantly better high-temperature properties, the ability of asphalt to resist rutting is improved, and the elastic recovery is increased [23]. Although these materials have improved various properties to different extent, their workability and stability are poor in cold weather, particularly during the winter snowfall season. This suggests that the performance enhancement of cold mix asphalt materials through PPA/SBS or PPA/SBR additives is limited. While the use of a single, PPA/SBS or PPA/SBR additives can partially address the problems of low strength and poor water stability, there is insufficient mention of whether the durability of cold mix asphalt materials is improved under aging conditions. Therefore, it is necessary to investigate the durability of cold patch materials under aging conditions.

In existing research, there are numerous evaluation methods for assessing the impact of multi-field coupling effects on the performance of asphalt mixtures. Guo analyzed the adhesion failure patterns of asphalt mixtures under temperature–humidity coupling conditions using digital image processing technology [24]. Yang investigated the aging characteristics of polymer-modified asphalt under temperature–irradiation coupling conditions through multi-scale morphological characterization techniques [25,26]. However, the diverse evaluation methods face limitations in their promotion and application processes [27,28,29,30,31]. Therefore, focusing on the stress–strain response of asphalt mixtures under multi-field coupling environments and exploring the viscoelastic evolution of these mixtures can provide precise quantitative measurements of the effects of multi-field coupling [32].

Viscoelasticity is one of the essential properties that reflect the stress–strain response of materials. In the study of asphalt mixtures, dynamic and static load tests are commonly employed to evaluate the linear viscoelastic behavior of the mixtures [1]. Under dynamic loading, specimens are typically cylindrical with dimensions of Φ100 mm × h150 mm, and their performance is assessed through dynamic modulus testing. Under static loading, creep or relaxation tests are usually conducted to maintain a constant stress or strain in the asphalt mixture, characterizing its creep and stress relaxation behavior under prolonged loading or relaxation conditions. Proposed predictive methods for the viscoelastic behavior of asphalt mixtures under aging conditions operate through statistical analysis and the development of empirical equations [33,34,35,36]. In the conversion and analysis of viscoelasticity under dynamic and static loads, extensively explored numerical methods are available for studying the conversion of viscoelastic properties under dynamic and static loading conditions, along with their accuracy in transformation [37,38,39,40,41,42,43].

GPC and FTIR are commonly used to analyze the composition and functional group changes in asphalt. Studies have shown that polymer-modified asphalt undergoes an increase in high molecular weight molecules and a decrease in low and medium molecular weight molecules after aging. During the aging process of SBS molecules, degradation into smaller molecules also occurs [4,5,7,44,45].

In summary, while scholars have conducted extensive research on the viscoelasticity of asphalt mixtures under aging conditions, the results have varied significantly, and there is a lack of microscopic analysis on the viscoelasticity of CMA. To address this gap, this study conducted indoor aging tests on CMA mixtures using RTFOT and PAV aging protocols to obtain CMA binders and mixtures at different stages of aging. Microscopic analysis was performed using FTIR and GPC to qualitatively and quantitatively analyze the characteristic functional groups and molecular weight of CMA. Additionally, rheological performance analysis included basic performance testing and temperature scanning tests. For the viscoelastic analysis of the mixtures, dynamic modulus testing was performed under dynamic loading conditions. Through the integration of microscopic analysis, rheological performance analysis, and viscoelastic analysis, this study reveals the evolution patterns of the viscoelasticity of CMA materials under various aging conditions.

## 2. Materials and Methods

### 2.1. Aging Tests

#### 2.1.1. RTFOT Test

The RTFOT origin is Hangzhou City, Zhejiang Province, China. The BA and CMA were placed on the aging tray to simulate short-time thermal oxidative aging of the asphalt (163 °C, 5 h). BA is denoted as ${BA}_{RTFOT}$ after the RTFOT, while CMA after the RTFOT is referred to as ${CMA}_{RTFOT}$.

#### 2.1.2. PAV Test

The PAV 20-h and PAV 40-h aging tests were carried out to simulate asphalt aging after 5–8 years and 8–12 years of service life, respectively [46], under experimental conditions (2.1 MPa, 100 °C). BA after 20 h and 40 h of PAV is designated as ${BA}_{PAV20}$ and ${BA}_{PAV40}$, respectively. Similarly, CMA after 20 h and 40 h of PAV is referred to as ${CMA}_{PAV20}$ and ${CMA}_{PAV40}$, respectively.

### 2.2. Asphalt and Asphalt Mixture

#### 2.2.1. Asphalt

**Figure 1:**
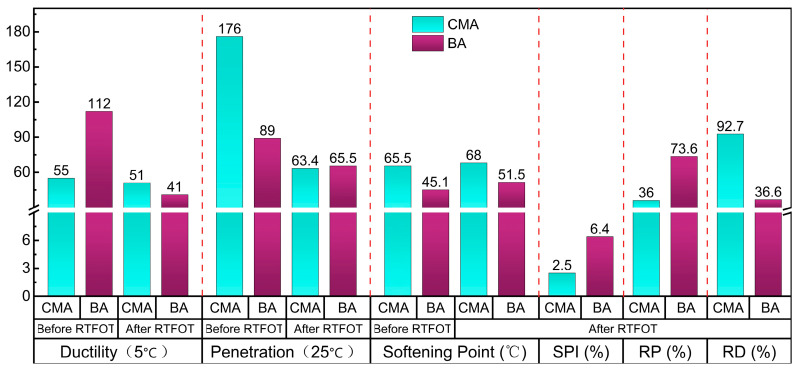
Main performance indexes of BA and CMA before and after RTFOT.

This study utilized one type of base asphalt (BA) and one type of CMA, PetroChina Karamay Petrochemical Co., Ltd. 90# (Grade A) road asphalt was chosen as the BA and served as the control group. Based on prior research [12], CMA was obtained by modifying BA. The results of the main performance indicators for the two types of asphalt before and after RTFOT are presented in Figure 1.

#### 2.2.2. Asphalt Mixture

##### Asphalt Mixture Design and Forming

Crushed stone and manufactured sand were sourced from Rongxiang Piez Stone Mine in Yanqi County, Xinjiang, China, while the mineral powder was produced by Shuo Zheng Mining Co., Ltd. in Hezhuo County, Xinjiang, China. The raw material testing results were conducted in accordance with the requirements of the Technical Standard for Testing Aggregates in Highway Engineering (JTG 3432-2024 [47]) and the Technical Specifications for Construction of Asphalt Pavements (JTG F40-2004 [48]). A summary of the test results for crushed stone, manufactured sand, and mineral powder is presented in Table 1 and Table 2.

The asphalt mixture proportions included two types of asphalt (BA, CMA) and two gradations (AC-13, AC-16). The road performance of the CMA mixture was evaluated through water stability tests, rutting tests, and low-temperature cracking resistance tests, and subsequently compared with that of the BA mixture. Two types of BA mixtures are denoted as ${BA}_{AC-13}$ and ${BA}_{AC-16}$, while CMA is referred to as ${CMA}_{AC-13}$ and ${CMA}_{AC-16}$.

**Figure 2:**
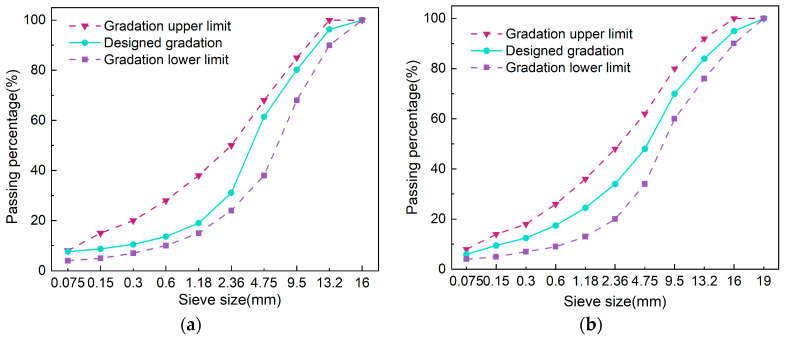
The designed gradation curves: (**a**) AC-13; (**b**) AC-16.

The asphalt mixture was initially formed using a rotary compactor to achieve the specimen specification (Φ150 mm × h170 mm). After demolding, the mixture was placed at room temperature for 48 h, then cored and cut into specimens (Φ100 mm × h150 mm) (Figure 2).

##### Asphalt Mixture Viscoelasticity Test

**Figure 3:**
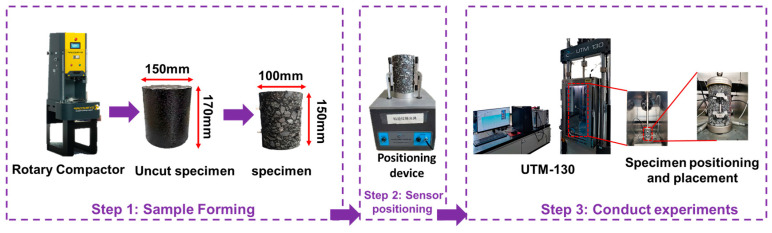
Dynamic modulus test.

The viscoelastic properties of the CMA mixture were tested using a UTM-130 testing machine. The dynamic modulus test was employed to characterize the viscoelastic behavior. Five experimental temperatures were used (−10, 4.4, 21.1, 37.8, and 54.4 °C), and six loading frequencies were used (0.1, 0.5, 1, 5, 10, and 25 Hz) [31,36]. The dynamic modulus test is a non-destructive testing method (Figure 3).

### 2.3. Dynamic Shear Rheometer (DSR) Test

DSR tests were analyzed using the CV0150 AR1500ex manufactured by TA Instruments, focusing primarily on the variations in $G^{*}$ and δ, as well as $G^{*}/sin\delta$.

### 2.4. Structural Characterization

#### 2.4.1. FTIR Test

FTIR was employed to analyze the functional groups of the binder before and after aging. The FTIR test was performed using the Nicolet iS20 Fourier-transform infrared spectrometer produced by Thermo Scientific in the USA. The infrared spectra wavenumber range was set as 400~4000 cm^−1^ (4 cm^−1^, 32 scans).

In order to quantitatively analyze the changes in the characteristic functional groups of rubber asphalt recycled binder before and after aging, the area of the C-H stretching vibration peaks within the ranges of 1325~1480 cm^−1^ and 2740~3000 cm^−1^ was used as a baseline. The carbonyl factor ($I_{C=O}$), sulfoxide factor ($I_{S=O}$), long-chain factor ($I_{LC}$), branched-chain factor ($I_{BC}$), aromatic factor ($I_{Aroma}$), and naphthenic factor ($I_{Alipha}$) are defined as follows, where the area A represents the integral of the infrared spectrum at the specified wavenumbers before and after aging [49].

(1) $$I_{C=O}=A_{1673.0-1718.5}/A_{1325.0-1480.0,\;2740.0-3000.0},$$

(2) $$I_{S=O}=A_{981.7-1057.0}/A_{1325.0-1480.0,\;2740.0-3000.0},$$

(3) $$I_{LC}=A_{704.0-734.8}/A_{1325.0-1480.0,\;2740.0-3000.0},$$

(4) $$I_{BC}=A_{1325.1-1392.6}/A_{1325.0-1480.0,\;2740.0-3000.0},$$

(5) $$I_{Aroma}=A_{1517.9-1637.5}/A_{1325.0-1480.0,\;2740.0-3000.0},$$

(6) $$I_{Alipha}=A_{1325.1-1392.6,1392.6-1479.4}/A_{1325.0-1480.0,\;2740.0-3000.0},$$

#### 2.4.2. GPC Test

Asphalt analysis was conducted using the Agilent 1260 Infinity II Gel Permeation Chromatograph and associated software manufactured by Agilent Technologies in the Santa Clara, CA, USA. Tetrahydrofuran (THF) was used as the mobile phase, and three chromatographic columns were connected in series to wash asphalt molecules based on their molecular sizes.

The chromatograms were divided into 13 segments and categorized into three groups, large molecule size compounds (LMS, sections 1–5), medium molecule size compounds (MMS, sections 6–9), and small molecule size compounds (SMS, sections 9–13), for further analysis of the compositional changes of the two types of asphalt before and after aging.

Asphalt molecules are often dispersed in nature. To characterize their molecular weight and molecular weight distribution, the commonly used measures include the number-average molecular weight ($M_{n}$), weight-average molecular weight ($M_{w}$), and polydispersity index (PDI). Here, $N_{i}$ represents the number of molecules with molecular weight $M_{i}$ (g/mol), while $W_{i}$ denotes the quantity of components with molecular weight $M_{i}$ (g/mol).

(7) $$M_{n}=\sum W_{i}/\sum W_{i}*M_{i-1},$$

(8) $$M_{w}=\sum W_{i}*M_{i}/\sum W_{i},$$

(9) $$\mathrm{PDI}=M_{w}/M_{n},$$

## 3. Results

### 3.1. Test Results of Asphalt Rheological Properties

The complex modulus ($G^{*}$) represents the capability of a material to resist shear deformation, while the phase angle (δ) signifies the ratio of viscous (non-recoverable) to elastic (recoverable) components. The $G^{*}$ aging index $C_{MAI}$ and δ aging index $P_{AAI}$ were used to evaluate the aging degree of the two types of asphalt. A higher $C_{MAI}$ and a lower P_AAI_ indicate more severe aging. The calculations for $C_{MAI}$ and $P_{AAI}$ are as follows:

(10) $$C_{MAI}=G^{*1}/G^{*2},$$

(11) $$P_{AAI}=\delta_{1}/\delta_{2},$$

Equation: $G^{*1}$, $G^{*2}$: $G^{*}$ before and after aging; $\delta_{1}$, $\delta_{1}$: δ before and after aging.

**Figure 4:**
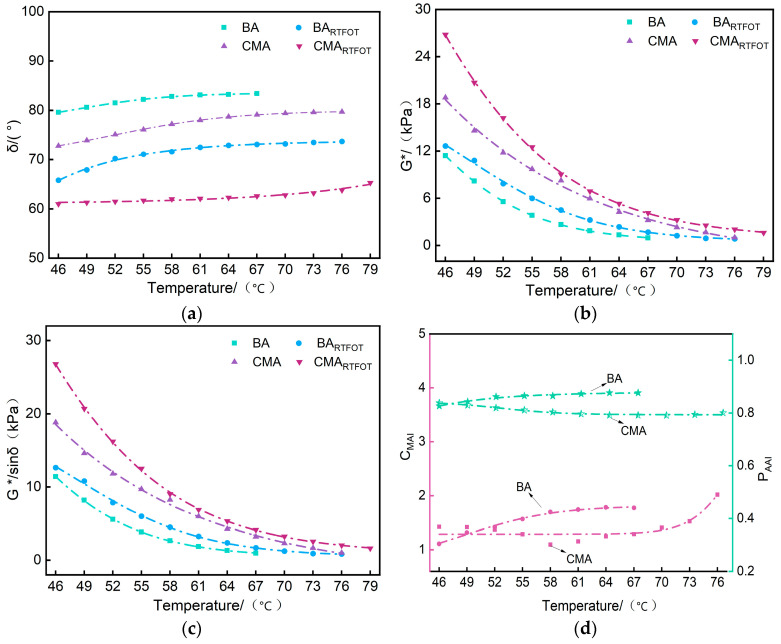
Rheological properties before and after RTFOT aging of BA and CMA. (**a**) δ; (**b**) $G^{*}$; (**c**) $G^{*}/sin\delta$; (**d**) $C_{MAI}$ and $P_{AAI}$.

Figure 4a–c depict the $G^{*}$, δ, and $G^{*}/sin\delta$ of BA and CMA before and after aging. It can be observed from Figure 4a–c that the $G^{*}$ of both asphalt samples decreases with increasing temperature. Contrastingly, the $G^{*}$ values of both asphalt samples significantly increased after RTFOT aging compared to the unaged samples, with CMA showing a more pronounced increase in $G^{*}$ values than BA. Following RTFOT aging, the δ curves of the two asphalt samples exhibited distinct trends. A reduction in δ signifies a decrease in the ratio of viscous to elastic components, indicating an increase in the stiffness of the asphalt. Post-RTFOT aging, BA demonstrated a higher δ compared to CMA

Figure 4d displays the changes in $C_{MAI}$ and $P_{AAI}$ for BA and CMA before and after aging. It is evident from Figure 4d that at the same testing temperature, CMA exhibited lower values within the range of 46–76 °C. After RTFOT aging, the BA $C_{MAI}$ ranged between 1.1 and 1.8, while CMA ranged between 1.1 and 2.0, with a more pronounced difference post-RTFOT aging. The trend for BA showed an initial increase followed by stabilization in $C_{MAI}$ with temperature variation. However, CMA was followed by an increase after 46 °C, indicating its superior aging resistance at high temperatures.

Figure 4d illustrates the changes in $P_{AAI}$ for BA and CMA before and after aging. It is evident from Figure 4d that BA overall exhibited lower $P_{AAI}$ values compared to CMA. Post-RTFOT aging, the $P_{AAI}$ values of BA slightly increased with temperature, whereas the $P_{AAI}$ values of CMA post-RTFOT aging did not strictly increase with temperature and showed a slight decrease within the 46–64 °C range, followed by a slight increase within the 64–76 °C range [16,50].

Based on the observed changes in $G^{*}$ and δ, it was determined that the degree of hardening for both asphalt types was greater following RTFOT aging than prior to aging. Both types of asphalt demonstrated strong long-term aging resistance, with CMA exhibiting superior aging resistance at elevated temperatures compared to BA. This observation is consistent with previous studies that suggest polymer-modified additives improve aging resistance.

### 3.2. FTIR Analysis

**Figure 5:**
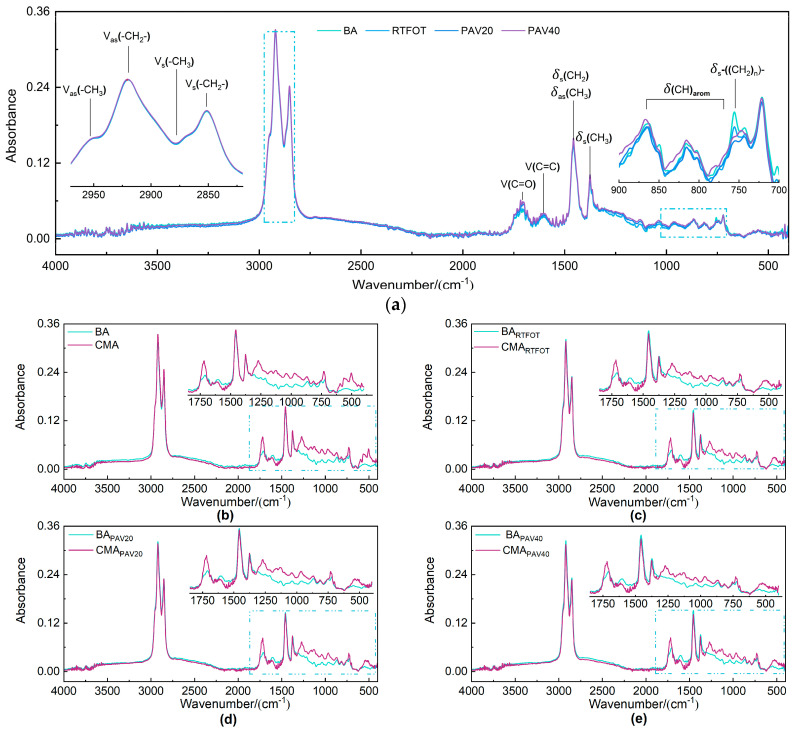
FTIR spectra of BA and CMA with different aging conditions. (**a**) FTIR spectra of BA and CMA with different aging conditions; (**b**) BA and CMA; (**c**) RTFOT; (**d**) PAV20; (**e**) PAV40.

To investigate the changes in viscoelastic properties of the two types of asphalt at the molecular structure level, FTIR spectroscopy was employed for quantitative analysis of the carbonyl index ($I_{c=o}$), sulfoxide index ($I_{s=o}$), long-chain index ($I_{LC}$), branched index ($I_{BC}$), aromatic index ($I_{ar}$), and aliphatic index ($I_{al}$) during the aging process of the asphalt (Figure 5).

The $I_{c=o}$ and $I_{s=o}$ indices were used to characterize the degree of aging of the asphalt, while the variations in $I_{LC}$, $I_{BC}$, $I_{ar}$, and $I_{al}$ were attributed to the evolution of the viscoelastic properties of the asphalt.

**Figure 6:**
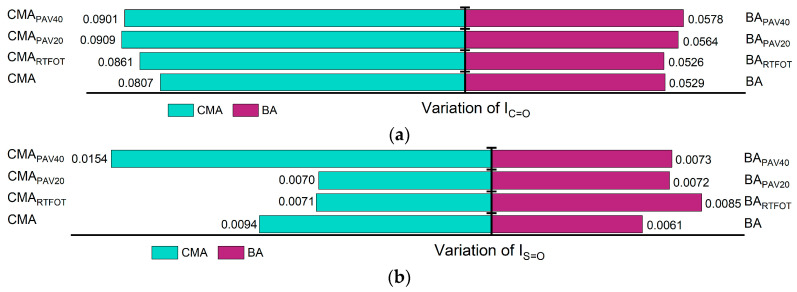
Changes in functional group indicators: (**a**) variation of $I_{c=o}$; (**b**) variation of $I_{s=o}$.

Figure 6 illustrates the trend of changes in the $I_{c=o}$ and $I_{s=o}$ as a function of aging degree. During the aging process, carbon oxidation occurs in the carbon atoms of the aromatic rings near the side chains, resulting in the formation of $I_{c=o}$, while the sulfur present in the asphalt undergoes oxidation to generate $I_{c=o}$. As the aging time increases, the oxidation reactions in the asphalt become more pronounced, leading to higher concentrations of $I_{c=o}$ and $I_{s=o}$. The test results and analyses presented in Figure 6 are consistent with these findings. Furthermore, the variations in $I_{c=o}$ and $I_{s=o}$ for the binders modified with SBS and SBR show a trend of initially decreasing followed by increasing, indicating a certain degree of mitigation of the aging process of the binder. This may be attributed to the absorption of lighter components by the SBS and SBR, indirectly slowing down the aging of the constituents within the asphalt. Additionally, SBS and SBR can gradually release these lighter components during the aging process, compensating for the components lost due to aging. The polymer chains in SBS and SBR also act to obstruct oxygen penetration into the asphalt, thereby reducing the rate of aging [51].

**Figure 7:**
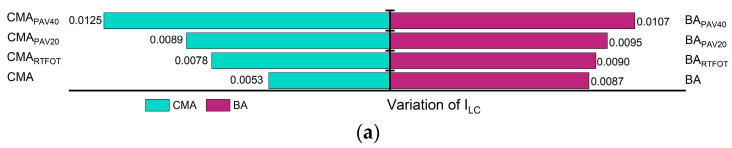

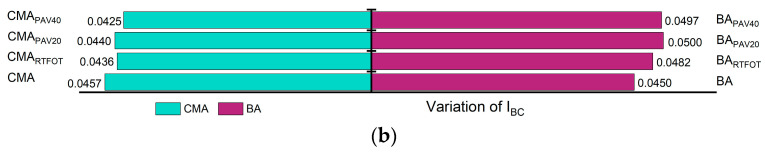
Changes in functional group indicators: (**a**) variation of $I_{LC}$; (**b**) variation of $I_{BC}$.

**Figure 8:**
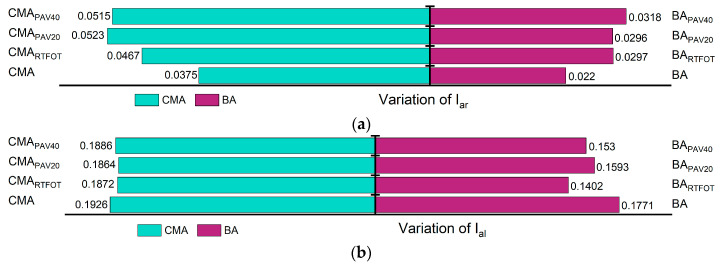
Changes in functional group indicators: (**a**) variation of $I_{ar}$; (**b**) variation of $I_{al}$.

In addition to the formation of $I_{s=o}$ and $I_{c=o}$, the aging process of asphalt is accompanied by condensation and addition reactions, wherein small molecules aggregate to form larger molecules. This leads to a reduction in the number of branched chains, resulting in longer molecular chains and an increased proportion of long chains (Figure 7). Figure 8 illustrates the trends of changes in the $I_{LC}$ and $I_{BC}$ during the aging process. With increasing aging severity, $I_{LC}$ rises while $I_{BC}$ decreases. For $I_{LC}$, the incorporation of SBS and SBR has a mitigating effect on its increase, indicating that the addition of SBS and SBR can effectively reduce the polymerization of long chains during the aging process. Conversely, the incorporation of SBS and SBR also slows down the reduction in $I_{BC}$, leading to an overall shortening of the asphalt molecular chains. As a result, the asphalt molecules with SBS and SBR additives exhibit greater flexibility compared to those without these modifiers.

During the aging process, the aliphatic components in asphalt gradually convert to aromatic compounds, resulting in a decrease in aliphatic content and an increase in aromatic content, as shown in Figure 8. The aromatic formation can be demonstrated as a combined result of two processes: the full hydrogenation of aromatic rings and the alkyl substitution of cycloalkanes. Notably, the incorporation of SBS and SBR slows the rate of reduction of aliphatic molecules during aging, thereby mitigating the aromatic conversion compared to binders without these additives. This indicates that during thermal oxidative aging, the absorption of lighter components by SBS and SBR effectively slows down part of the aromatic conversion process of aliphatic molecules. Furthermore, the long polymer chains of SBS and SBR provide effective barriers against the further penetration of oxygen, thereby reducing the conversion of aliphatic to aromatic compounds [51,52].

**Figure 9:**
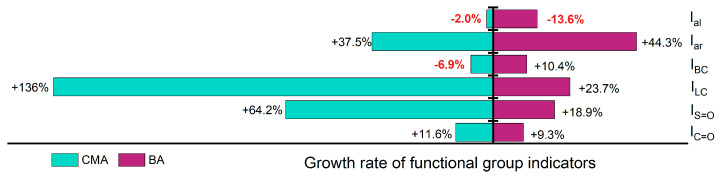
Changes in functional group indicators at different aging stages.

Figure 9 illustrates the trends in changes of $I_{c=o}$, $I_{s=o}$, $I_{LC}$, $I_{BC}$, $I_{ar}$, and $I_{al}$ before and after aging. The $I_{ar}$ of BA decreased by 13.6%, while the $I_{ar}$ of CMA also decreased by 2%. Additionally, the $I_{BC}$ of CMA decreased by 6.9%. The growth rates of the other parameters for both BA and CMA before and after aging exceeded 9.3%, with CMA displaying a remarkable increase of 136% in $I_{LC}$.

### 3.3. GPC Analysis

**Figure 10:**
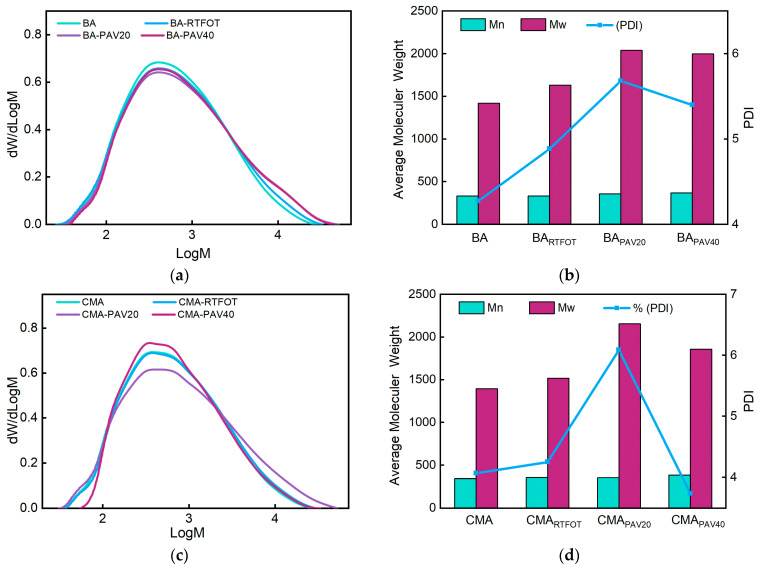
GPC results of BA and CMA: (**a**) $M_{w}$ distribution of BA; (**b**) $M_{w}$ of BA of different aging conditions; (**c**) $M_{w}$ distribution of CMA; (**d**) $M_{w}$ of CMA of different aging conditions.

By using GPC, the changes in number-average molecular weight ($M_{n}$), weight-average molecular weight ($M_{w}$), and polydispersity index (PDI) of BA and CMA samples before and after aging with RTFOT, PAV20, and PAV40 were obtained and are depicted in the curve shown in Figure 10. Studies have shown that $M_{w}$ is sensitive to compounds with high molecular weights; a higher $M_{w}$ indicates a higher amount of high-molecular-weight substances. On the other hand, $M_{n}$ is sensitive to compounds with low molecular weights; a larger $M_{n}$ indicates a higher amount of low-molecular-weight substances. The polydispersity coefficient PDI represents the distribution of both high- and low-molecular-weight substances, with a larger PDI indicating a more dispersed distribution of all molecules [53,54,55].

Figure 10a,c show the $M_{w}$ distribution of BA and CMA during the aging process. The *x*-axis represents the logarithmic weight-average molecular weight of asphalt, while the *y*-axis represents the relative content of asphalt. Figure 10a,c demonstrate that after RTFOT aging, PAV20 aging, and PAV40 aging, the overall molecular weight of asphalt shifted towards larger molecules. During aging, the lighter components in asphalt volatilize and transform into colloids and asphaltenes. Aging ultimately leads to a decrease in the content of lighter components in asphalt and an increase in the content of asphaltenes. Asphaltenes are high-molecular-weight substances. Consequently, after aging, the molecular weight distribution curve of asphalt gradually shifts to the right, indicating a reduction in polymer molecular weight due to the degradation of long-chain polymers [56].

Figure 10b,d reveal that after RTFOT aging and PAV20 aging, BA and CMA show varying degrees of increase in $M_{n}$, $M_{w}$, and PDI values, indicating an increase in molecular weight and dispersity of aged asphalt. This indicates a reduction in low $M_{w}$ components and an increase in high $M_{w}$ components following RTFOT and PAV20 aging, which enhances the shear resistance of aged asphalt and improves its high-temperature performance. The polydispersity coefficient PDI increases for both asphalt types after RTFOT aging and PAV20 aging, indicating a more dispersed molecular distribution after these aging processes. However, after PAV40 aging, the $M_{n}$, $M_{w}$, and PDI values of BA and CMA show varying degrees of decrease, mainly due to degradation of long-chain polymers during the aging process [53].

**Figure 11:**
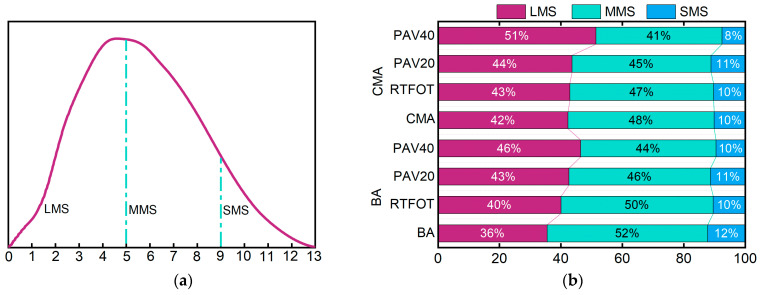
Changes in molecular weight of BA and CMA before and after aging: (**a**) division of LMS, MMS, and SMS; (**b**) molecular weight distribution results.

Figure 11 shows that before aging, the content of LMS in base asphalt was 36%, MMS content was 52%, and SMS content was 12%. The content of LMS, MMS, and SMS in CMA was 42%, 48%, and 10%, respectively. During the aging process, both types of asphalt showed a decrease in MMS and SMS content, with an increase in LMS content. This was due to oxidation reactions during aging, leading to the conversion of non-polar components into polar components, as well as the evaporation of SMS at high temperatures, resulting in a reduction of lower molecular weight components in the asphalt [4,57,58]. This is corroborated by the analysis in Figure 11. After RTFOT aging, the content of LMS, MMS, and SMS in base asphalt was 40%, 50%, and 10%, respectively, while for CMA it was 43%, 47%, and 10%. Following PAV20 aging, the content of LMS, MMS, and SMS in BA was 43%, 46%, and 11%, and for CMA it was 44%, 45%, and 11%. After PAV40 aging, the content of LMS, MMS, and SMS in BA was 46%, 44%, and 10%, and in CMA it was 51%, 41%, and 8%. The molecular weight of base asphalt and CMA increased after RTFOT aging and PAV aging. This indicates that the aging level of asphalt is significantly more severe after PAV aging than RTFOT aging, and CMA exhibits superior resistance to long-term hot oxygen aging. The decrease in peak values of the SBS and SBR molecular curve after aging suggests a reduction in the number of SBS and SBR molecules after aging, indicating that the SBS and SBR molecules have already played a role and delayed the aging process of modified asphalt, resulting in an overall increase in asphalt molecular mass [57]. The changes in asphalt molecular weight lead to an increase in the complex modulus of asphalt at the macro level, consistent with the changes in rheological properties of aged asphalt in this experiment [2].

The aforementioned study reveals that after aging, the number of high-molecular-weight molecules increases in CMA, while the number of low and medium-molecular-weight molecules decreases [2,3,4,57].

### 3.4. High and Low Temperature Performance of Asphalt Mixture

#### 3.4.1. Marshall Test

**Figure 12:**
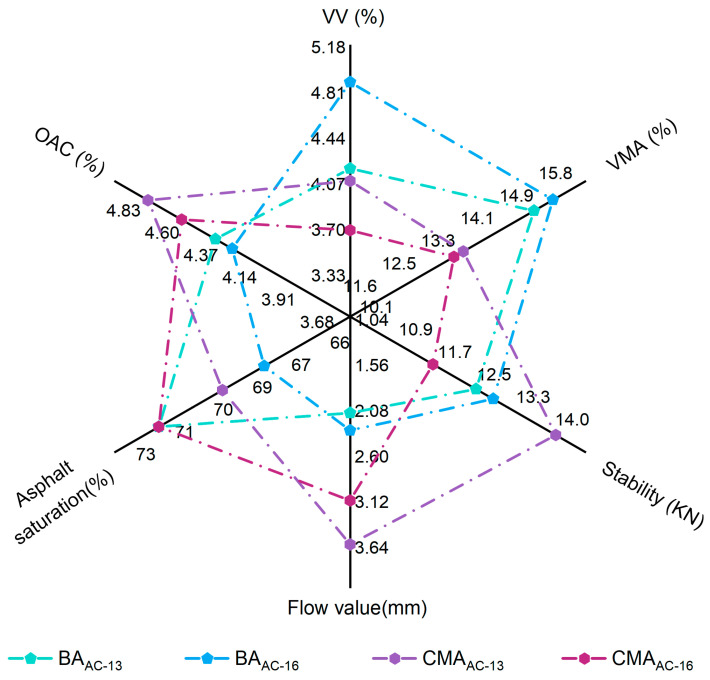
Marshall test results.

The Marshall test results for the optimal asphalt-to-aggregate ratios of the ${BA}_{AC-13}$, ${BA}_{AC-16}$, ${CMA}_{AC-13}$, and ${CMA}_{AC-16}$ asphalt mixtures are presented in Figure 12.

As shown in Figure 12, the voids ratio of the asphalt mixture specimen (VV), aggregate voids ratio of the asphalt mixture specimen (VMA), optimal asphalt content (OAC), and the incorporation of SBS and SBR increased the stability value and OAC value of the BA mixture, decreasing the VV value and VMA value. As previously mentioned, this is attributed to the addition of polymers, which enhances the adhesion of BA, thereby reducing the stability values [41,42,44] and increasing the maximum load that the CMA mixture can bear, which is reflected in the stability value. Additionally, an increase in asphalt content may lead to a higher proportion of free asphalt, which could also decrease the Marshall stability value of the asphalt mixture.

#### 3.4.2. Analysis of Road Performance

A comparative performance analysis was conducted between the CMA mixture and the BA mixture, focusing on their high-temperature stability (dynamic stability (DS)), low-temperature cracking resistance (bending tensile strength ($R_{B}$); maximum bending tensile strain ($\varepsilon_{B}$), flexural stiffness modulus ($S_{B}$)), water stability (residual stability ($M_{S0}$), and freeze–thaw splitting strength ratio (TSR)), among other road performance characteristics.

**Figure 13:**
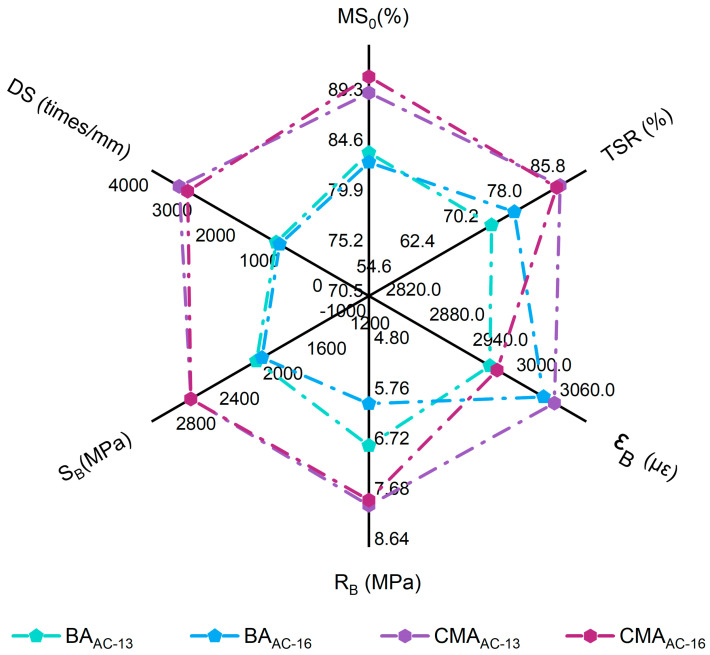
Results of the analysis of road performance indicators.

Figure 13 presents the results of road performance tests for four types of asphalt mixtures. The DS values for the four mixtures were 1135, 1055, 3365, and 3172 times/mm, all of which meet the highest requirements specified in the JTGE20-2011 standard [59], namely, greater than 800 times and 2800 times/mm. The results indicate that the polymer network crosslinking structure inherent in the CMA system enhances the stiffness and high-temperature stability of the CMA mixture, resulting in excellent high-temperature rutting resistance.

The $R_{B}$ of ${CMA}_{AC-16}$ is 1.3 times that of ${BA}_{AC-16}$. Additionally, the $\varepsilon_{B}$ for the four asphalt mixtures is ranked from smallest to largest as follows: ${BA}_{AC-13}$ < ${BA}_{AC-16}$ < ${CMA}_{AC-13}$ < ${CMA}_{AC-16}$. The results indicate that the low-temperature cracking resistance of the CMA mixtures is superior to that of the BA mixtures. The incorporation of SBS and SBR has improved the low-temperature performance of the CMA mixtures, achieving an enhancement of 133% compared to the BA mixtures, thereby meeting the highest requirement specified in the standards, which is greater than 2800.

The $M_{S0}$ and the TSR for all four asphalt mixtures meet the standard’s highest requirements, specifically, residual stability greater than 85% and a TSR greater than 80%. The $M_{S0}$ value for ${CMA}_{AC-16}$ is notably higher, exceeding that of ${BA}_{AC-16}$ by 10%. Furthermore, a comparison of TSR indicates that the $M_{S0}$ of CMA asphalt mixtures is significantly greater than 10% in the BA mixtures. These results demonstrate that CMA mixtures exhibit excellent water stability, and the addition of SBS and SBR significantly enhances the adhesion and aggregate coating performance of CMA. This, in turn, reduces the susceptibility of the modified CMA asphalt system to moisture and improves the resistance of the CMA mixtures to water damage and frost.

### 3.5. Viscoelasticity

For the dynamic modulus ($E^{*}$) of the four types of asphalt mixtures, an improved sigmoid model was used to establish the master curve of $E^{*}$ at the reference temperature [34,38]. The sigmoidal model is expressed as follows in Equation (12):

(12) $$lg\left|E^{*}\right|=\varphi +\alpha /\left(1+e^{\beta +\left(\gamma {lgf}_{r}\right)}\right),$$

Equation: |$E^{*}$| is the dynamic modulus, MPa; φ is the logarithm of the minimum value of the $E^{*}$; α is the logarithm of the difference between the maximum and minimum $E^{*}$ values; β and γ are parameters describing the shape of the master curve. The ${lgf}_{r}$ as shown in Equation (13):

(13) $${lgf}_{r}=lgf+{lg\alpha }_{T},$$

where ${lgf}_{r}$ is the reduced frequency at the reference temperature, Hz; ${lg\alpha }_{T}$ is the shift factor, a function of temperature T; and *f* is the frequency, Hz.

**Figure 14:**
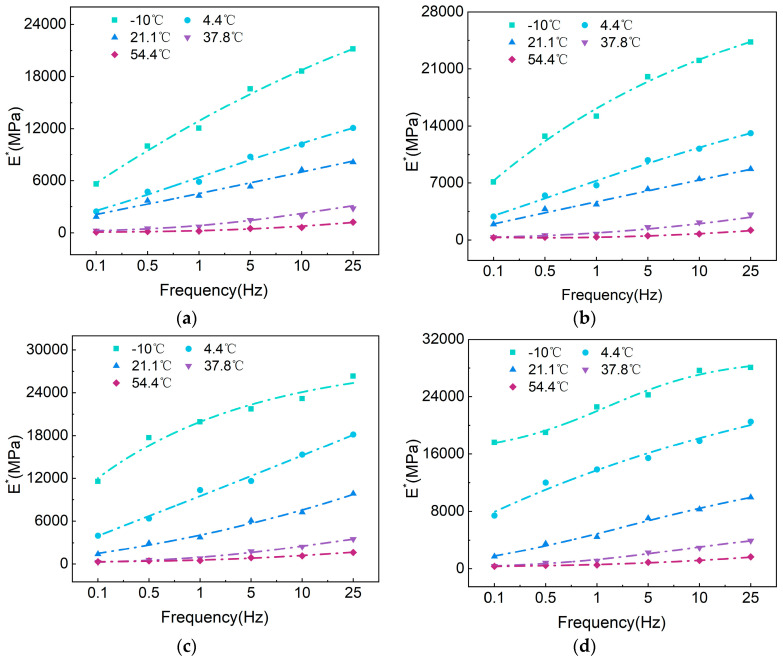
$E^{*}$: (**a**) ${BA}_{AC-13}$; (**b**) ${BA}_{AC-16}$; (**c**) ${CMA}_{AC-13}$; (**d**) ${CMA}_{AC-16}$.

**Figure 15:**
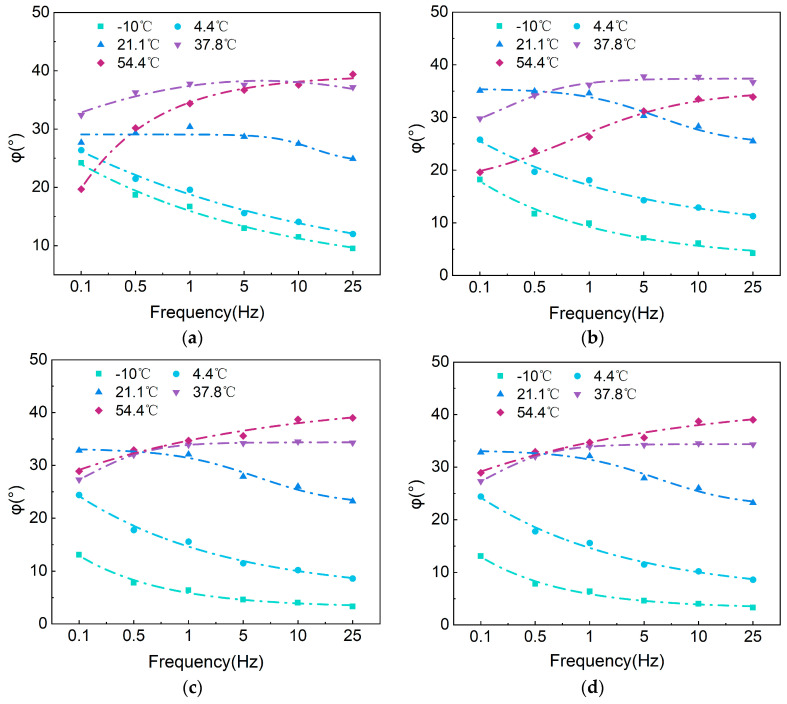
φ: (**a**) ${BA}_{AC-13}$; (**b**) ${BA}_{AC-16}$; (**c**) ${CMA}_{AC-13}$; (**d**) ${CMA}_{AC-16}$.

Figure 14 and Figure 15 show that the $E^{*}$ of asphalt mixtures gradually increase with frequency at the same temperature, while at the same frequency, the $E^{*}$ decreases as temperature rises. As the temperature increases, the elasticity of the asphalt mixture weakens, and its viscosity enhances, causing the material properties to lean more towards viscous behavior. Consequently, the $E^{*}$ of the asphalt mixture decreases, making it less capable of resisting permanent deformation. Under dynamic loading conditions, the simultaneous incorporation of SBS and SBR into CMA mixtures increases the dynamic modulus in the 25 Hz and −10 °C range by 24.3% (AC-13) and 15.4% (AC-16), and reduces the φ by 55.8% (AC-13) and 40% (AC-16).

Taking ${CMA}_{AC-16}$ as an example, the variation of $E^{*}$ with temperature exhibits a significant nonlinear characteristic. Taking 0.5 Hz as an example, the $E^{*}$ value at 54.4 °C is only 8.6% of that at 21.1 °C. As the temperature increases (or the frequency decreases), the asphalt mixture softens and approaches a more viscous state, resulting in a decrease in $E^{*}$ and an increase in the phase angle δ. Under high-temperature conditions (or low-frequency conditions), the influence of asphalt on the mixture diminishes; at this point, the primary factor driving the modulus variation of the asphalt mixture is the interlocking force of the aggregates. Further increases in temperature (or reductions in frequency) lead to a decrease in φ, as well as a reduction in $E^{*}$, although the values begin to stabilize.

At the same temperature and frequency, the $E^{*}$ of BA is lower than that of CMA, and the change rate of the $E^{*}$ of the CMA mixture in response to temperature is smaller than that of BA. In the low-frequency range of 0.1 Hz to 1 Hz, there is a significant increase in the $E^{*}$ values; however, this increase becomes more gradual between 1 Hz and 25 Hz. This is mainly because, at higher loading frequencies, the asphalt pavement approaches elastic deformation, preventing further increases in the $E^{*}$. Between −10 °C and 21.1 °C, the $E^{*}$ of the asphalt mixture decreases rapidly, indicating that the $E^{*}$ is considerably affected by temperature in the low-temperature range, while the impact diminishes after 21.1 °C.

Analyzing the variation of $E^{*}$ with temperature (frequency) reveals that at higher temperatures (or lower frequencies), the $E^{*}$ value decreases and approaches the minimum value $E_{min}^{*}$. Conversely, at lower temperatures (or higher frequencies), the $E^{*}$ value increases and approaches the maximum value $E_{max}^{*}$. The generalized logarithmic sigmoidal model effectively captures the variation characteristics of $E^{*}$.

**Figure 16:**
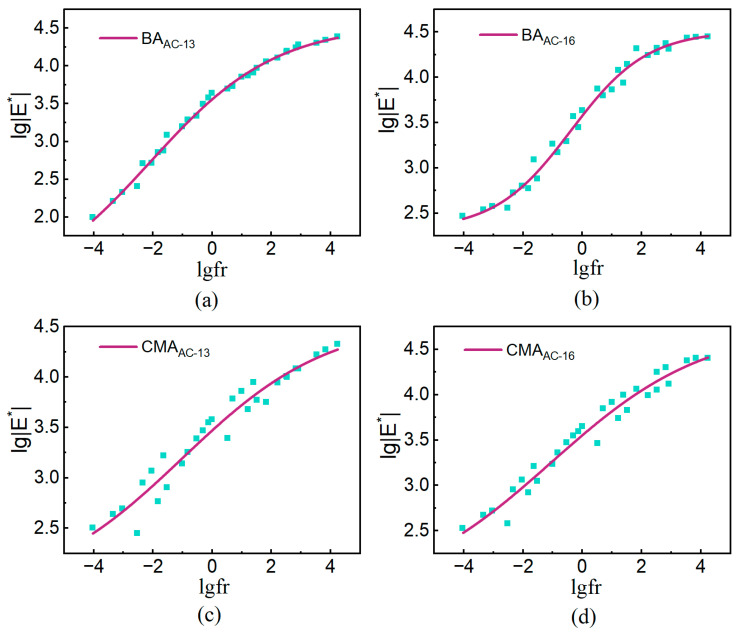
Master curve of $E^{*}$: (**a**) ${BA}_{AC-13}$; (**b**) ${BA}_{AC-16}$; (**c**) ${CMA}_{AC-13}$; (**d**) ${CMA}_{AC-16}$.

The sigmoid model demonstrates a good fitting performance, with an average coefficient of determination ($R^{2}$) exceeding 0.9445. Figure 14, Figure 15 and Figure 16 illustrate the $E^{*}$, φ, and master curve of $E^{*}$. As the test frequency decreases, the phase angle initially increases and then decreases, indicating the presence of an inflection point in the master curve. This behavior is attributed to the greater viscosity of the binder at low frequencies and high temperatures, where the aggregate skeleton responds to external stress and strain. As the test frequency gradually increases, the binder exhibits more elastic behavior under low-temperature and high-frequency conditions, leading to a gradual decrease in phase angle and an increase in dynamic modulus. The generalized logarithmic sigmoidal model can be utilized to compute the master curve of dynamic viscoelastic parameters for the CMA mixture, enabling a comprehensive expression of the dynamic mechanical characteristics of the CMA mixture.

## 4. Conclusions

This paper investigates the viscoelastic evolution of CMA materials through microscopic analysis, rheological testing, and the viscoelastic analysis of mixtures, leading to the following key conclusions:

(1) In terms of molecular structure, the findings indicate that the incorporation of SBS and SBR increases the levels of carbonyl and sulfoxide factors while reducing the level of long-chain factors, which slows the rate of change in large molecule content and enhances the aging resistance of the asphalt. Regarding molecular weight components, the addition of SBS and SBR reduces the rate of change of LMS by more than 6%, while also mitigating the rate of change in overall molecular weight distribution to below 50%.

(2) Under dynamic loading conditions, the simultaneous incorporation of SBS and SBR into CMA mixtures increases the dynamic modulus in the 25 Hz and −10 °C range by 24.3% (AC-13) and 15.4% (AC-16), and reduces the φ by 55.8% (AC-13) and 40% (AC-16).

(3) This study clarifies the evolution patterns of the characteristic functional groups and molecular weight of CMA. However, there remain limitations in revealing the viscoelastic evolution of the binder from a compositional perspective. Future research could conduct a four-component analysis of CMA, quantitatively characterizing the contents of asphaltenes, aromatics, saturated fractions, and resins to evaluate the impact of compositional changes on the viscoelastic properties of CMA.

## Figures and Tables

**Table 1:** Test results of crushed stone, machine-made sand, and mineral powder.

Detecting Parameter	Apparent Relative Density	Relative Density of Gross Volume	Water Absorption (%)	Detection Result
Sample Name				
Gravel (15–20) mm	2.871	2.842	0.28	Qualified
Gravel (10–15) mm	2.862	2.827	0.43	
Gravel (5–10) mm	2.855	2.803	0.64	
Gravel (3–5) mm	2.849	2.794	0.69	
Machine sand (0–3) mm	2.821	2.755	/	
Mineral powder	2.718	/	/	
Technical requirements	Gravel ≮ 2.60	/	≯2.0	/
	Machine sand ≮ 2.50			

**Table 2:** Test results of mineral powder.

Limestone	Property	Unit	Tested Values	Code Values
Mineral powder	Apparent density(kg·m^−3^)	g/cm^3^	2.718	≥2.50
	Water carrying capacity (%)	%	0.104	≤1
	Passing percentage (%)	0.6 mm	100	100
		<0.15 mm	98.1	90~100
		<0.075 mm	87.2	75~100
	Appearance	/	No agglomerates	No agglomerates
	Hydrophilic coefficient	/	0.4	<1
	Plasticity index (%)	%	2.56	<4

## Data Availability

The original contributions presented in this study are included in the article. Further inquiries can be directed to the corresponding author.
